# Self-assembly into virus–like particles of the recombinant capsid protein of porcine circovirus type 3 and its application on antibodies detection

**DOI:** 10.1186/s13568-019-0940-0

**Published:** 2020-01-07

**Authors:** Yu Wang, Gang Wang, Wei-Tong Duan, Ming-Xia Sun, Meng-Hang Wang, Shang-Hui Wang, Xue-Hui Cai, Ya-bin Tu

**Affiliations:** State Key Laboratory of Veterinary Biotechnology, Harbin Veterinary Research Institute, Chinese Academy of Agriculture Sciences (CAAS), Harbin, 150069 China

**Keywords:** PCV3, VLPs, Diagnostic, Swine, ELISA

## Abstract

PCV3 capsid protein (Cap) is an important antigen for diagnosis and vaccine development. To achieve high-level expression of recombinant PCV3 Cap in *Escherichia coli* (*E. coli*), the gene of wild-type entire Cap (wt-eCap) was amplified from clinical samples, and three optimized entire Cap (opti-eCap) and one optimized Cap deleted nuclear location signal (NLS) (opti-dCap) gene fragments encoding the same amino acid sequence with wt-eCap were synthesized based on the codon bias of *E. coli*. Those gene fragments were inserted into the pET30a expression vector. One recombinant strain with the highest expressed soluble eCap from four entire Cap (one wt-eCap and three opti-eCap) and one recombinant strain expressed opti-dCap were selected for further purification. The purified eCap and dCap were identified by transmission electron microscopy (TEM), a large number of round hollow particles with a diameter of 10 nm virus-like particles (VLPs) were observed in eCap, whereas irregular aggregation of proteins observed in dCap. After formation the VLPs were applied as a coating antigen to establish an indirect ELISA (I-ELISA) for detection of PCV3-specific antibody in swine serum. 373 clinical swine serum samples from China collected in 2019 were tested utilizing the VLP-based I-ELISA method under optimized conditions. To the best of our knowledge, this is the first report of self-assembly into VLPs of PCV3 recombinant Cap. Our results demonstrated that the VLP-based I-ELISA will be a valuable tool for detecting the presence of PCV3 antibodies in serum samples and will facilitate screening of large numbers of swine serum for clinical purposes.

## Introduction

Porcine circovirus (PCV) has three strains with the following nomenclature PCV1, PCV2 and PCV3. PCV1 was first isolated from a contaminated porcine kidney PK-15 cell line and has no pathogenicity for pigs. PCV2 can cause clinical symptoms including postweaning multisystemic wasting syndrome (PMWS), pneumonia, porcine dermatitis, nephropathy syndrome (PDNS), and reproductive failure (Madson and Opriessnig 2011). PCV3 was identified as a new member of PCV in 2015 (Palinski et al. 2017), additional studies showed that PCV3 appeared in other countries (China, Poland, Brazil, Korea, Denmark, Italy and Spain) (Franzo et al. 2018a; Kim et al. 2018b; Shen et al. 2018; Stadejek et al. 2017; Tochetto et al. 2018). Specifically, the existence of PCV3 in pigs can be traced back to as early as 1996 in China according to early studies (Sun et al. 2018). Although the pathogenicity of PCV3 is currently debated, studies have shown that it has the potential to threaten the swine industry directly (Jiang et al. 2019) as well as to enhance pig susceptibility to other microorganisms (Chen et al. 2019; Li et al. 2018). Currently, the common difficulty for the study of PCV3 is that the virus cannot be isolated from cell lines. However, unraveling the characteristic of the virus is still ongoing by constructing virus-like particles (VLPs) based on genetic engineering technology which can be achieved without the virus. These VLP-based studies may provide new ideas leading to a better understanding of the PCV virus which will lead to potential treatments and diagnotics.

VLPs are self-assembled macromolecules composed of viral structural proteins but lacking of genetic materials. Due to the increased safety and ease of use VLPs are preferable for development of serologic diagnostic tests. The enzyme-linked immunosorbent assay (ELISA) based on VLPs is widely used to measure antibodies or neutralizing epitopes (Almanza et al. 2008; Chao et al. 2019). For example, PCV2 VLPs are used as coating antigens to be able to detect serum neutralizing antibodies (SNAbs) in ELISA (Nainys et al. 2014; Zhang et al. 2016). Currently, PCV3 Cap proteins harvested from insect cells or *E. coli* have been used as antigens to detect PCV3-specific antibodies in an ELISA (Deng et al. 2018; Zhang et al. 2019). There is still lacking a VLP-based serological diagnosis method for PCV3.

In this study, one recombinant strain with the highest expressed soluble opti-eCap was selected and successfully defined conditions for the protein to self-assemble into VLPs. The VLPs were purified through a two-step chromatographic technology. Additionally, a highly specific, sensitive and reproducible VLP-based indirect ELISA (I-ELISA) has been established. This new PCV3 ELISA is a valuable tool for detecting the presence of PCV3 antibodies in serum samples and will facilitate the screening of large numbers of swine serum for clinical purposes.

## Materials and methods

### Swine serum samples

Positive sera was collected from clinical swine serum samples. To assess the specificity of the assay, samples were screened for only PCV3 infection, excluding other pathogens such as PCV2 and PRRSV by PCR and ELISA. The samples with the highest concentration were selected as positive serum and used to coat ELISA plates for the PCV3 VLP ELISA and used for western blot and ELISA optimization. Negative serum samples were collected from forty pathogen-free (SPF) piglets which were obtained from the Experimental Animal Center at the Veterinary Research Institute (Harbin, China). 373 clinical serum samples including 60 wild boar serum samples were collected from China in 2019 for testing using the PCV3 VLP ELISA.

### Gene amplification

**Fig. 1:**
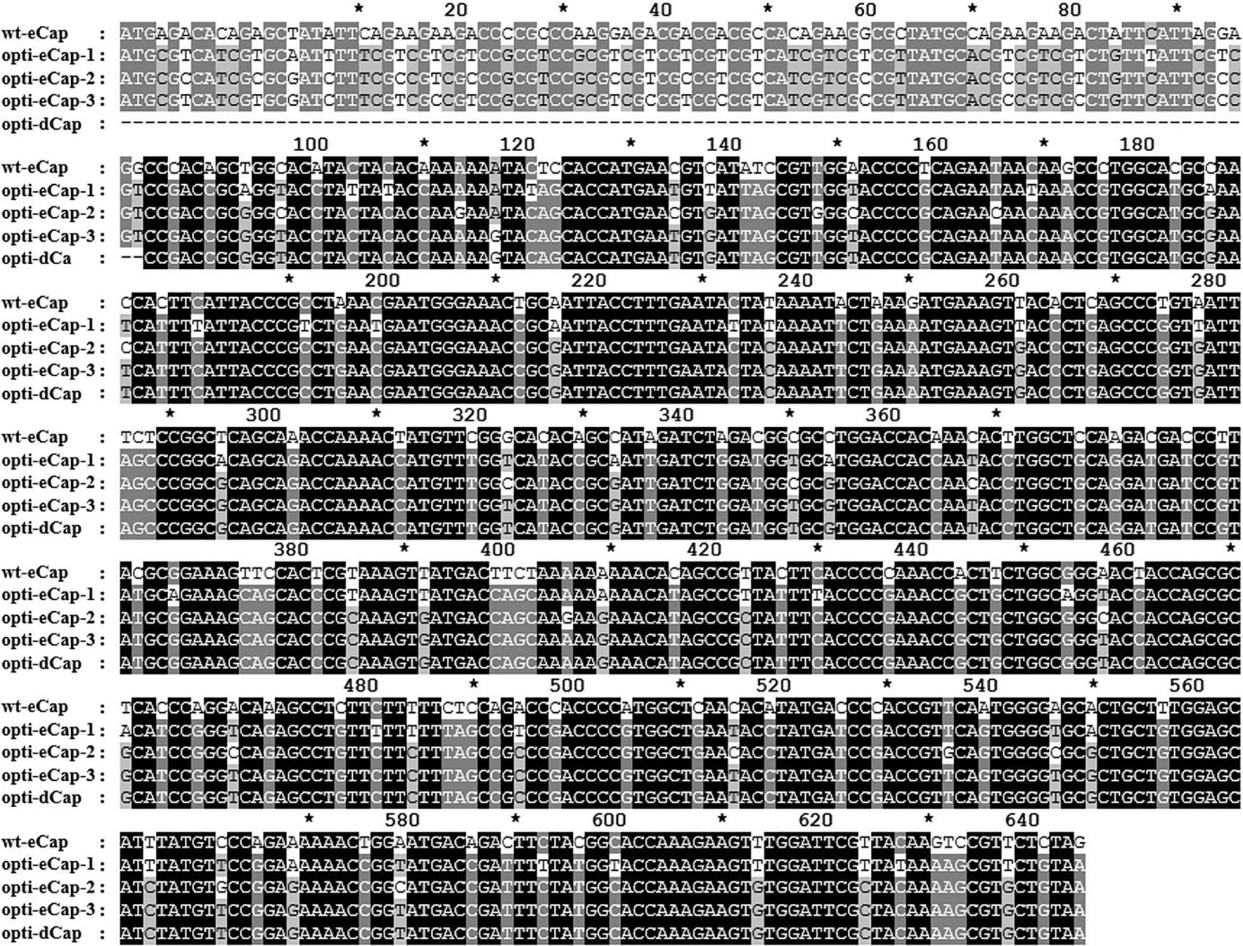
Nucleotide sequence alignment between the wild-type and optimized Cap genes. Full-length Cap of PCV3 (645 bp) was optimized for *E. coli* codon usage

The wt-eCap was amplified by PCR using DNA from lymph nodes of postweaning multisystemic wasting syndrome-suffering pigs. Opti-eCap-1 was fully optimized for the full-length gene of PCV3 Cap protein based on factors such as codon bias and GC content, while opti-eCap-2 and opti-eCap-3 were partially optimized. Meanwhile, one optimized Cap deleted nuclear location signal (NLS) (opti-dCap) gene fragments encoding the same amino acid sequence with wt-eCap were synthesized, and a 6 × His-tag was fused to the NH_2_-terminal end of the dCap to aid protein purification. Sequence alignment of the four entire Cap (one wt-eCap and three opti-eCap) and opti-dCap is provided in Fig. 1.

### Construction and expression of recombinant Cap protein in *E. coli*

One wt-eCap, three opti-eCap and one opti-dCap gene fragments were subcloned into the pET30a expression vector via the NdeI/XhoI sites. The recombinant fusion proteins eCap and dCap were obtained by transforming the corresponding plasmid into *Escherichia coli (E. coli)* BL21 (DE3) under conditions of 220 rpm shaking speed at 37 °C until the OD_600_ reached 0.5, at which time 0.4 mM isopropyl-β-d-thiogalactopyranoside(IPTG) was added and the bacteria were incubated at 25 °C for 20 h. Bacteria were harvested by centrifugation at 6000×*g* for 10 min at 4 °C. The cell pellet was resuspended in 40 ml of 50 mM Tris–HCl buffer (pH 8.0) and sonicated on ice for 300 cycles of 3 s pulses at 6 s intervals using a Cell Ultrasonic Crusher (Cole Parmer, USA) at 39% amplitude. Lysates were divided into supernatant and pellet by centrifugation at 12,000×*g* for 20 min at 4 °C. Pellets were resuspended in PBS at a volume equal to the supernatant. Expression and solubility of eCap and dCap were analyzed by sodium dodecyl sulfate polyacrylamide gel electrophoresis (SDS-PAGE) and Western blot. Briefly, equal molar amounts of each recombinant protein were separated using 12% SDS–PAGE. Separated proteins were transferred electrophoretically onto a polyvinylidene difluoride (PVDF) membrane. Unbound sites on the membrane were blocked with blocking buffer at overnight. The membrane was incubated with specific pig positive serum for 1 h, and was washed in PBST (PBS containing 0.05% of Tween 20) three times. Next, anti-Pig IgG (whole molecule)-FITC antibody produced in goat (SIGMA, USA, 1:10,000) was added and incubated for 1 h. The final colorimetric reaction was developed at room temperature using the Infrared Imaging Systems (GE, USA).

### Purification of VLPs

The highest expressed soluble opti-eCap-3 was chosen and purified by anion-exchange chromatography as the first step. The supernatant was loaded on a DEAE Bestarose Fast Flow column (Bestchrom, China) in an automated FPLC system (AKTA, GE-Healthcare Life Sciences, USA). After the column had been washed with 50 mM Tris–HCl buffer (pH 8.0), eCap was eluted and collected with buffer B (50 mM Tris and 150 mM NaCl, pH 8.0). The purity of the eCap protein was assessed by SDS-PAGE. Formation of PCV3 VLPs was verified with TEM (H7650, HITACHI, Japan). Then, the product was subjected to size-exclusion chromatography equipped with a prepacked Sepharose 6FF 16/96 column (Bestchrom, China) in buffer B. The flow rate was set to 1.5 ml/min and the first peak was collected, and VLPs were detected by SDS-PAGE and TEM. In addition, the recombinant dCap was purified by Ni–NTA affinity (GE, USA) and also detected by TEM.

### Standardization of the indirect PCV3 VLP-ELISA procedure

Purified PCV3 VLPs were used as antigens for development of an indirect ELISA (I-ELISA) to detect anti-PCV3 antibodies in swine serum. The optimal dilutions of antigen and serum were determined by a checker board titration with positive and negative swine sera. The concentration of PCV3 VLPs were measured by BCA (Thermo, USA). The prepared antigen was used to coat 96-well ELISA plates (BIOFIL, China) ranging from 0.5, 1, 2.5, 5, 7.5 to 10 μg/ml and 100 μl per well diluted in carbonate–bicarbonate buffer (pH 9.6). Sera diluted in PBS ranging from 1:50, 1:100, 1:150 to 1:200 (v/v) was used and tested to determine the optimal serum dilution. The optimal dilutions of antigen and serum were determined on the basis of the maximum difference in absorbance at 450 nm between positive and negative serum (P/N) were selected on a larger scale. In addition, the reaction temperature, time and other conditions were also optimized.

### PCV3 VLP-ELISA procedure

After optimization, 100 μl of 5 μg/ml PCV3 VLPs in carbonate–bicarbonate buffer was coated onto a 96-well ELISA microplate (Corning, USA) at 4 °C overnight. The antigen-coated plate was washed three times with PBST and blocked with PBST containing 5% (w/v) skim milk at 37 °C for 3 h. After washing three times, 100 μl of diluted serum samples were added and incubated at 37 °C for 1 h. The plates were washed three times with PBST, followed by incubation for 1 h at room temperature with 100 μl diluted HRP-labeled goat anti-pig IgG (Solarbio, China, 1:5000). After being washed three times, the plates were incubated with 100 μl tetramethylbenzidine (TMB, Solarbio) in the dark for 15 min at room temperature, which was used as a chromogenic substrate. Reactions were stopped by adding 50 μl of ABTS stop solution (2 M HCl) and the absorbance (450 nm) was measured using an ELISA plate reader (PE, USA).

### Determination of cut-off value

Forty negative sera were used to determine the cut-off value. All sera were subjected to PCV3 VLP-ELISA three times to abate deviation. The mean OD450 nm value (X) and standard deviation (SD) were calculated, and the cut-off value was defined as X + 3SD.

### Reproducibility and cross-reactivity assay

A total of 12 serum samples were selected to evaluate the reproducibility of the PCV3 VLP-ELISA. For each sample, the coefficient of variation (CV) was calculated between plates (inter-assay variation) and within the same plate (intra-assay variation). Each sample was tested in five different plates on different occasions to determine the inter-assay CV and five replicates within each plate were used to calculate the intra-assay CV. For cross-reactivity assay, standard positive sera of PCV2, PRRSV, PEGV, TEGV, PRV and CSFV were tested in triplicate according to PCV3 VLP-ELISA procedure and the OD values were calculated. In addition, six PCV3 positive and one negative serum were used to determine assay sensitivity.

### Accession numbers of synthetic sequences

For gene optimization, Optimum-Gene™ algorithm was used to produce a single gene which is highly expressed. All sequences have been uploaded to GenBank, wt-eCap (Gene Accession Number MN310686), opti-eCap-1 (Gene Accession Number MN714691), opti-eCap-2 (Gene Accession Number MN714692), opti-eCap-3 (Gene Accession Number MN714693) and opti-dCap (Gene Accession Number MN714694).

## Results

### Preparation of PCV3 VLPs from *E. coli*

**Fig. 2:**
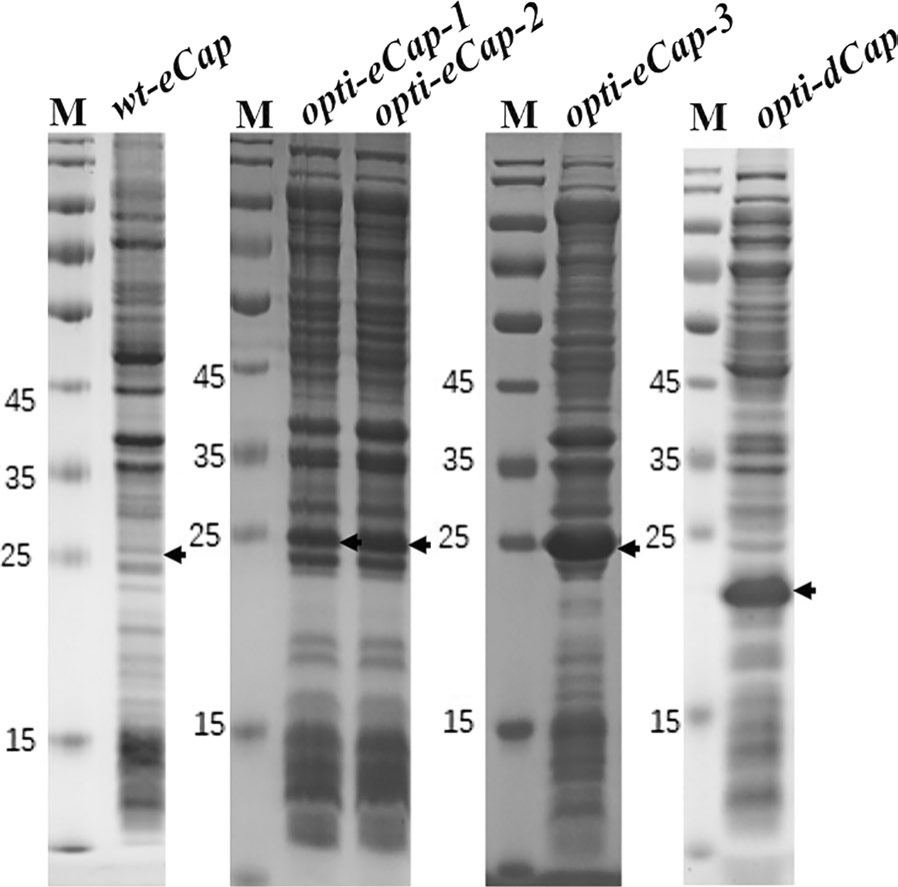
Preparation of eCap and dCap produced in *E. coli*. SDS-PAGE of protein preparation and staining with coomassie blue

**Fig. 3:**
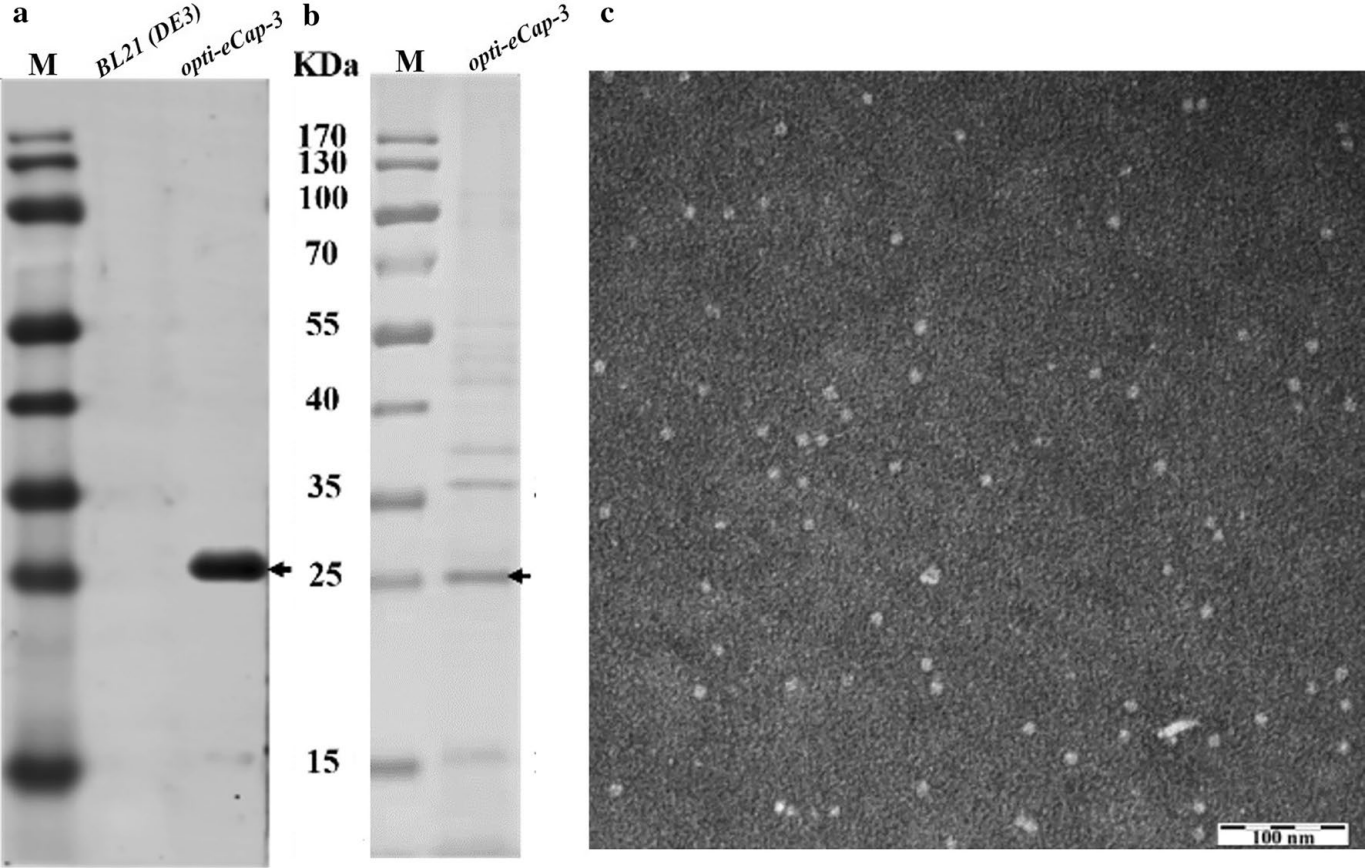
Analysis and purification of opti-eCap-3. **a** Western blots with swine PCV3-specific positive serum. **b** SDS-PAGE result and **c** TEM images of opti-eCap-3 purified by ion exchange chromatography

**Fig. 4:**
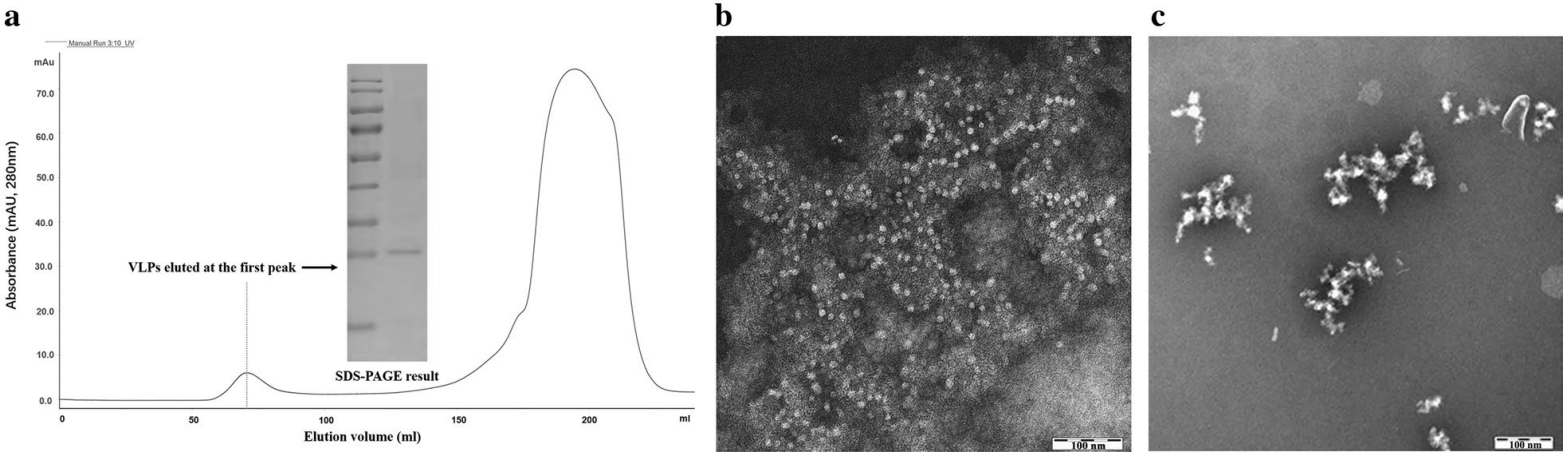
Characterization of PCV3 VLPs by size-exclusion chromatography. **a** Gel filtration profile of PCV3 VLPs. The VLPs were eluted at the first peak and subjected to SDS-PAGE and **b** TEM. **c** TEM images of purified opti-dCap-3

The recombinant expression plasmids eCap-pET30a and dCap-pET30a were constructed for efficient expression of target proteins in *E. coli*. Recombinant Cap protein was harvested by induction with 0.4 mM IPTG and incubation at 25 °C for 20 h with shaking. The results of SDS-PAGE showed wt-eCap (molecular weight ~ 25 kDa) was hardly expressed in *E. coli*, while opti-eCap (molecular weight ~ 25 kDa) can be expressed in a considerable level and opti-eCap-3 was dominant (Fig. 2). For the future research, the highest expressed soluble opti-eCap3 was selected and it can also specifically react with positive serum (Fig. 3a). Due to the complex composition of the supernatant, host proteins or subcellular organelles, it is impossible to see clear viral particles directly under the TEM (data not shown). Therefore, purification by chromatography is necessary. During the purification process, higher purity was preferred over yield rate. After the purification by IEC, SDS-PAGE results showed little heteroprotein bands (Fig. 3b), 10 nm particles can be clearly observed under the TEM (Fig. 3c). However, there were still some special-shaped impurities detected in the particles. In order to make the results more clear and useful for subsequent ELISA establishment, the purest sample had been obtained from the next SEC (Fig. 4a). Uniform particles without any impurities were observed under TEM (Fig. 4b). A large fraction of the particles were in an aggregate state. Therefore, we can confirm the expression of the opti-eCap successfully self-assembled into virus-like particles. Furthermore, the recombinant eCap(molecular weight ~ 21 kDa) also had a high level of expression in *E. coli* (Fig. 2) but there were only irregular aggregation of proteins observed in TEM (Fig. 4c). It means that removing the NSL may lose the ability of VLPs forming.

### Standardization of the PCV3 VLP-ELISA procedure

**Fig. 5:**
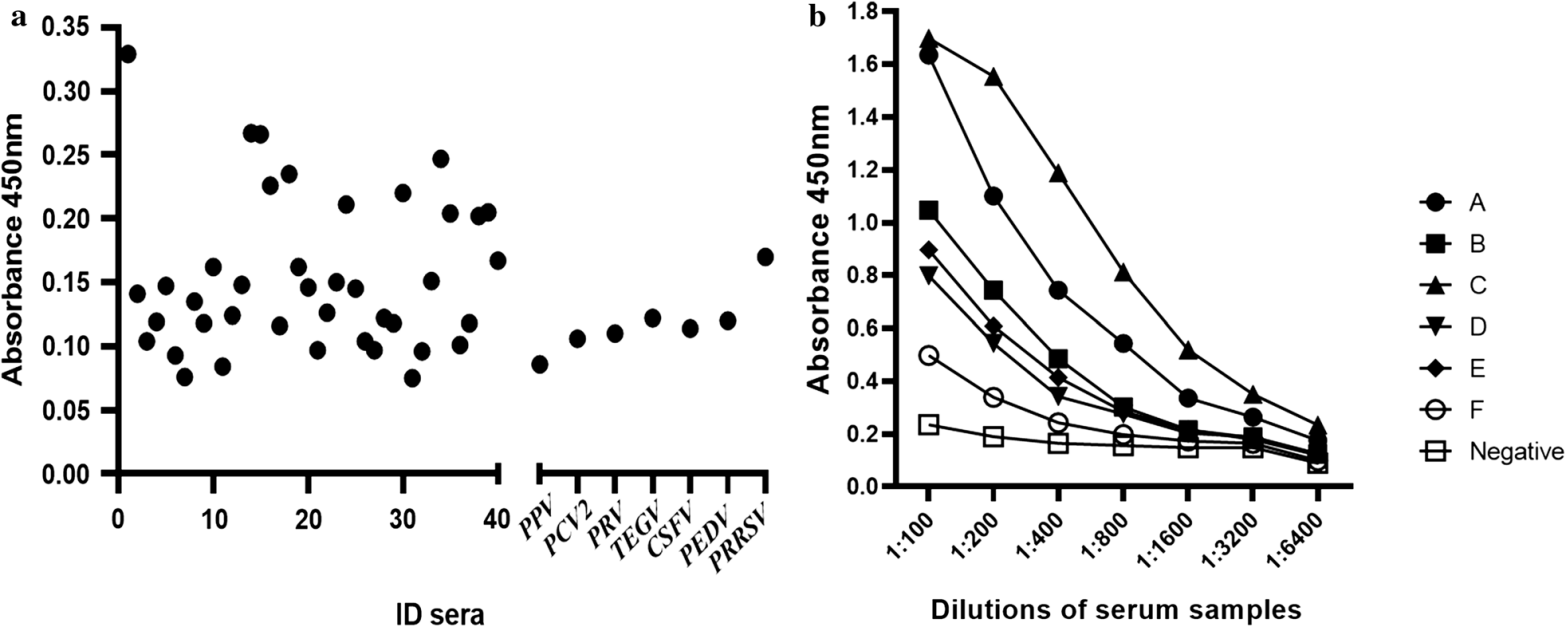
**a** Determination of the cutoff value and specificity for VLP ELISA. **b** Determination of the sensitivity of VLP ELISA

Optimization conditions of the VLP-based I-ELISA were determined by OD450 nm and P/N values. 50 mM carbonate–bicarbonate buffer (pH 9.6) and 5% skimmed milk was chosen for the optimal coating and blocking buffers, respectively. The checkerboard titration tests indicated that optimal antigen concentration for plate coating was 5 µg/ml with a serum dilution of 1:200 in a maximal P/N ratio, and all experiments were performed in triplicates (Table 1). 40 negative serum samples were employed for deciding cut-off value, the mean OD value and SD was 0.154 and 0.059, respectively using in the result (Fig. 5a). Hence, the cut-off value of the VLP ELISA was calculated as 0.331 (mean + 3SD). Any serum with OD450 greater than or equal this threshold was regarded as positive, otherwise it was determined to be PCV3 antibody negative.

**Table 1:** Optimal dilutions for antibody and coating antigen for VLP-based I-ELISA

Serum dilution	Concentration of coating antigen (X ± SD, µg/ml)					
	10.0	7.5	5.0	2.5	1.0	0.5
50 × (+)	2.646 ± 0.016	2.338 ± 0.006	1.645 ± 0.023	1.46 ± 0.059	1.1 ± 0.022	0.838 ± 0.019
50 × (−)	0.305 ± 0.014	0.3 ± 0.087	0.234 ± 0.112	0.174 ± 0.006	0.132 ± 0.015	0.112 ± 0.069
P/N	8.675	7.793	7.030	8.391	8.333	7.482
100 × (+)	2.088 ± 0.003	1.797 ± 0.015	1.552 ± 0.029	1.104 ± 0.084	0.832 ± 0.048	0.669 ± 0.001
100 × (−)	0.181 ± 0.075	0.166 ± 0.006	0.147 ± 0.098	0.12 ± 0.069	0.095 ± 0.003	0.085 ± 0.004
P/N	11.536	10.825	10.558	9.200	8.758	7.871
150 × (+)	1.72 ± 0.067	1.524 ± 0.036	1.339 ± 0.052	0.962 ± 0.026	0.756 ± 0.036	0.576 ± 0.006
150 × (−)	0.155 ± 0.057	0.132 ± 0.019	0.122 ± 0.022	0.094 ± 0.068	0.082 ± 0.047	0.077 ± 0.024
P/N	11.097	11.545	10.975	10.234	9.220	7.481
200 × (+)	1.604 ± 0.006	1.376 ± 0.017	1.338 ± 0.011	1.079 ± 0.047	0.777 ± 0.033	0.584 ± 0.005
200 × (−)	0.126 ± 0.044	0.109 ± 0.025	0.102 ± 0.097	0.097 ± 0.014	0.081 ± 0.074	0.093 ± 0.016
P/N	12.730	12.624	13.118	11.124	9.593	6.280

All non-PCV3 serum samples (PPV, PCV2, PRV, TEGV, PEDV, CSFV, and PRRSV) were negative as the result of VLP ELISA (Fig. 5a). The OD450 nm values decreased with successive dilutions of six positive serum samples indicating high sensitivity (Fig. 5b).

### Antibody detection using the PCV3 VLP-ELISA

**Fig. 6:**
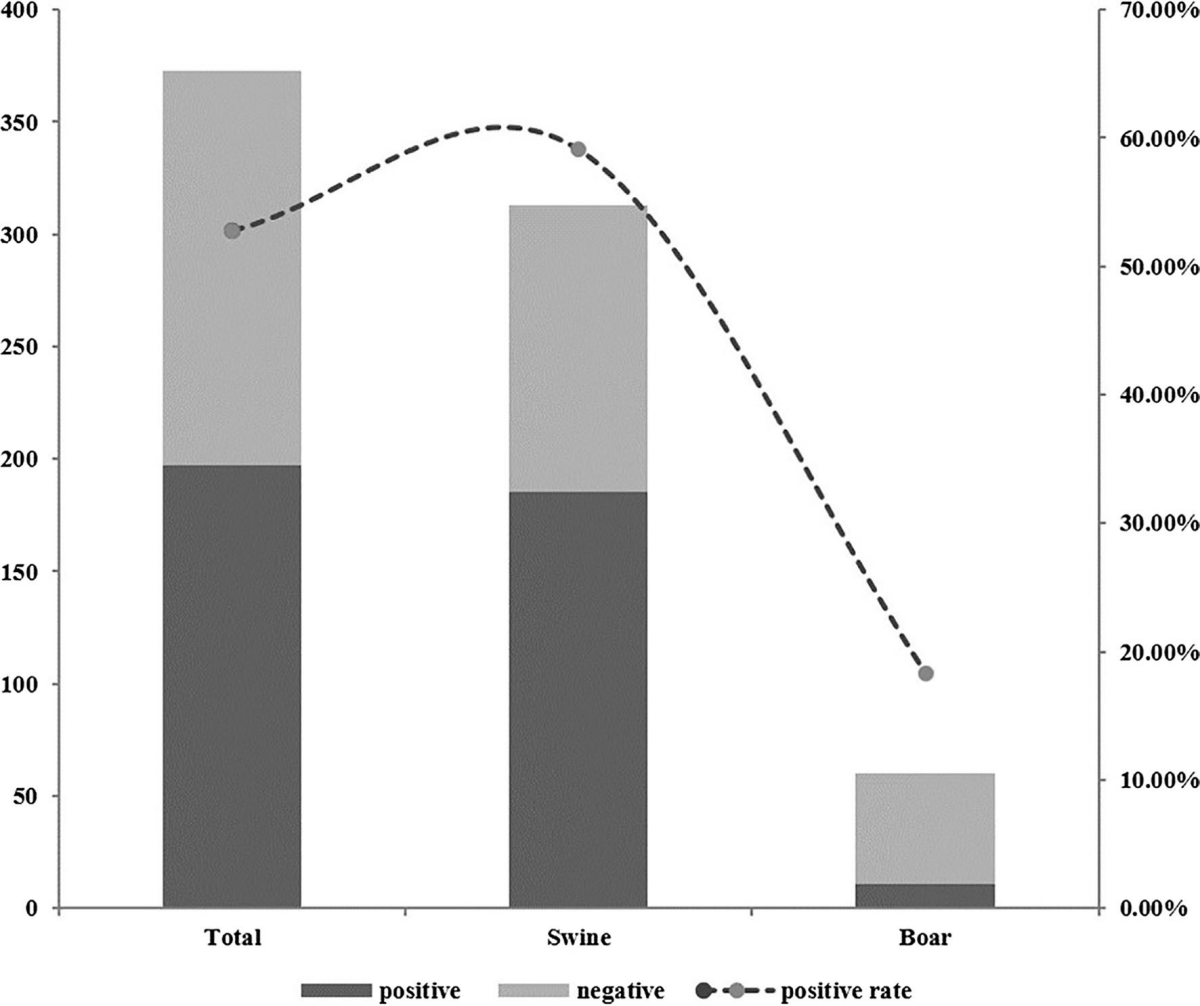
A VLP ELISA was used to determine PCV3 positive rate of swine serum samples (n = 373), and used to compare the positive rate of swine and wild boars

Swine serum from China collected in 2019 were tested. In total, 197 samples (52.82%) tested positive by VLP ELISA. It was worth noting that the positive rate of swine (59.11%) is much higher than the wild boar (18.33%) (Fig. 6). This suggests that differences between species and feeding conditions may affect PCV3 infection. In conclusion, the VLP ELISA has high application value in the future epidemiology research of PCV3.

## Discussion

VLPs, composed of viral structural proteins without genetic materials, are self-assembled macromolecules. Because of their ability in stimulating strong immune response and plentiful antibody production, they are regarded as candidates of novel vaccines. (Garcea and Gissmann 2004). VLPs provide more safety in use and the possibility of large scale production of vaccines with reproducible high quality results compared with traditional live-attenuated or inactivated virus vaccines (Noad and Roy 2003; Raghunandan 2011). Some VLPs, such as Hepatitis B surface antigen (HBsAg) VLPs, human papillomavirus (HPV) VLPs and Malaria VLP-base vaccines have already been clinically used for the prevention of infectious diseases (Kim and Kim 2017a). In recent years, the technology of expressing capsid protein (Cap) of PCV2 and self-assembly into virus-like particles (VLPs) has advanced, and can be used in multiple recombinant protein expression systems including baculovirus, yeast and *E. coli* (Liu et al. 2008; Nainys et al. 2014; Wu et al. 2016). Many commercial vaccines based on VLPs have effectively prevented the infection of PCV2 (Beach and Meng 2012). Similar to PCV2, there are two major open reading frames (ORFs), ORF1 and ORF2, in PCV3 genome. ORF2 encode the immunogenic Cap which is the sole structural protein of the viral coat (Palinski et al. 2017). Whether the idea of forming recombinant Cap (rCap) into VLPs is equally applicable to PCV3 is the main purpose in our research.

In this study, The *E. coli* expression system has been employed successfully for expression of PCV3 VLPs due to its relative simplicity, low cost, and fast high-density cultivation, which is significant in future diagnosis and vaccine development. However, the N-terminal NLS domain of the Cap protein is abundant in arginine residues and contains several rare codons for *E. coli* that impedes a foreign gene expression, which is disadvantageous for full-length Cap expression (Wu et al. 2012). Using codon optimization can overcome the difficulty of high-level expression of full-length Cap. Removing of NLS has also been utilized to improve the expression efficiency and stability of expressed protein in *E. coli* but has failed to self-assemble into VLPs.

The diameter of the circovirus is around 17 nm, similar to beak and feather disease virus (BFDV), bat circovirus (BtCV) and PCV2. The morphological study of PCV3 has not be conducted, whether 10 nm particles is the true size of the virus remains to be determined. Previous studies have shown that expressing the full-length PCV3 cap gene and NLS domains presenting within the N-terminal arginine rich motif (ARM) may cause misfolding of the protein and induce formation of circular virus complexes of 10–12 nm (Sarker et al. 2016). Other groups have published that, different sizes of VLPs appear in different expression systems (Bucarey et al. 2009; Kim et al. 2018a; Wu et al. 2016), a number of factors including storage conditions (ion-strength, pH and etc.) as well as the process design also influence the characteristics of VLPs (Effio and Hubbuch 2015; Fernandes et al. 2013; Kim and Kim 2017b). 10 nm particles can be used as a preliminary evaluation standard before the real virus morphology of PCV3 is discovered.

Hydrophobic interaction chromatography (HIC) has also been used as a step in the purification process. But eCap cannot be eluted under any conditions even if the least hydrophobic filler was selected. Therefore, it is suspected that PCV3 VLPs are highly hydrophobic. This conclusion can also be derived from genetic analysis as the entire Cap gene contains many hydrophobic amino acids. Therefore high-purity particles exist in an aggregated form under the TEM, which also explained the observation that the target protein eluted at the first peak of sepharose 6FF column. Theoretically 10 nm particles should elute at the end of this gel. Virions have properties that tend to aggregate (Gerba and Betancourt 2017), the aggregate status may be changed by adjustment of the pH or salt concentration and type of cationic salt in suspension (Mattle et al. 2011). VLPs may also encounter the problem of aggregation due to their large size and complex structure, this can occur during the process such as during separation, purification and storage (Chen et al. 2015; Jezek et al. 2009; Kissmann et al. 2008; Shi et al. 2005).

VLPs are preferable for development of serologic diagnostic tests, as they mimic the structure of virus with repetitive surface epitopes of viral antigens in a proper conformation compared to monomeric viral candidate antigens. The enzyme-linked immunosorbent assay (ELISA) based on VLPs is widely used to measure antibodies or the neutralizing epitopes (Almanza et al. 2008; Chao et al. 2019). PCV2 VLPs as coating antigens can detect serum neutralizing antibodies (SNAbs) in ELISA (Nainys et al. 2014; Zhang et al. 2016). Some reports show that PCV3 infection in pigs do not present any significant clinical signs or symptoms and wild boars may also have susceptibility (Franzo et al. 2018b; Klaumann et al. 2019; Zheng et al. 2017), thus it is particularly important to establish an effective antibody detection method to assess the PCV3 infection in pigs.

The isolation and cultivation of PCV3 is a recognized problem in the world. There is a report that the virus can be rescued from an infectious PCV3 DNA clone (Jiang et al. 2019). Our lab is actively trying to rescue the virus through the method reported in this article. Without virus it is impossible to carry out immunization and challenge experiments to prove whether the VLPs have protective effect on PCV3 infection as a vaccine because the virus has not been successfully isolated to date. To provide another solution here we describe the establishment of a VLP-based I-ELISA for antibody detection. In this process, the choice of positive serum was extremely important. In addition to PCV3, the specific positive serum cannot be detected with other pathogens by PCR and ELISA. Active viremia indicates that the virus is in the infection stage. The VLPs can react specifically with such serum as the result of Western and ELISA experiments, which fully demonstrates the immunogenicity for PCV3.

As a novel swine virus, positive results either through PCR or ELISA, are required since they indicate recent infection with PCV3. ELISA has more advantages due to its less operation labor and higher throughput, which can rapidly confirm the PCV3 infection status in pig populations. The VLP ELISA showed good sensitivity to positive serum samples and had no cross reactivity with other swine viral pathogens including PCV2. Subsequently, the VLP ELISA was successfully applied to the antibodies detection of 373 clinical serum samples from China collected in 2019, indicating that this method can be more widely applied to the epidemiological research of PCV3.

In conclusion, this is the first report of the ability of PCV3 VLPs to self-assemble which were successfully expressed in *E. coli* and applied in the development of an ELISA for testing the specific antibodies of clinical pig serum. The VLP ELISA was highly specific, sensitive and reproducible, it is a valuable tool to monitor the prevalence of the PCV3 virus. The invention of virus-like particles will play a significant role in providing a new tool for the study of PCV3.

## Data Availability

All the relevant data and materials are published in the article.

## References

[CR1] Almanza H, Cubillos C, Angulo I, Mateos F, Caston JR, van der Poel WH, Vinje J, Barcena J, Mena I (2008). Self-assembly of the recombinant capsid protein of a swine norovirus into virus-like particles and evaluation of monoclonal antibodies cross-reactive with a human strain from genogroup II. J Clin Microbiol.

[CR2] Beach NM, Meng XJ (2012). Efficacy and future prospects of commercially available and experimental vaccines against porcine circovirus type 2 (PCV2). Virus Res.

[CR3] Bucarey SA, Noriega J, Reyes P, Tapia C, Saenz L, Zuniga A, Tobar JA (2009). The optimized capsid gene of porcine circovirus type 2 expressed in yeast forms virus-like particles and elicits antibody responses in mice fed with recombinant yeast extracts. Vaccine.

[CR4] Chao DY, Whitney MT, Davis BS, Medina FA, Munoz JL, Chang GJ (2019). Comprehensive evaluation of differential serodiagnosis between zika and dengue viral infections. J Clin Microbiol.

[CR5] Chen Y, Zhang Y, Quan C, Luo J, Yang Y, Yu M, Kong Y, Ma G, Su Z (2015). Aggregation and antigenicity of virus like particle in salt solution—a case study with hepatitis B surface antigen. Vaccine.

[CR6] Chen N, Li S, Ye M, Huang Y, Huang Y, Xiao Y, Yu X, Dong J, Tian K, Zhu J (2019). A novel NADC30-like porcine reproductive and respiratory syndrome virus (PRRSV) plays a limited role in the pathogenicity of porcine circoviruses (PCV2 and PCV3) and PRRSV co-infection. Transbound Emerg Dis.

[CR7] Deng J, Li X, Zheng D, Wang Y, Chen L, Song H, Wang T, Huang Y, Pang W, Tian K (2018). Establishment and application of an indirect ELISA for porcine circovirus 3. Arch Virol.

[CR8] Effio CL, Hubbuch J (2015). Next generation vaccines and vectors: designing downstream processes for recombinant protein-based virus-like particles. Biotechnol J.

[CR9] Fernandes F, Teixeira AP, Carinhas N, Carrondo MJ, Alves PM (2013). Insect cells as a production platform of complex virus-like particles. Expert Rev Vaccines.

[CR10] Franzo G, Legnardi M, Hjulsager CK, Klaumann F, Larsen LE, Segales J, Drigo M (2018). Full-genome sequencing of porcine circovirus 3 field strains from Denmark, Italy and Spain demonstrates a high within–Europe genetic heterogeneity. Transbound Emerg Dis.

[CR11] Franzo G, Tucciarone CM, Drigo M, Cecchinato M, Martini M, Mondin A, Menandro ML (2018). First report of wild boar susceptibility to Porcine circovirus type 3: high prevalence in the Colli Euganei Regional Park (Italy) in the absence of clinical signs. Transbound Emerg Dis.

[CR12] Garcea RL, Gissmann L (2004). Virus-like particles as vaccines and vessels for the delivery of small molecules. Curr Opin Biotechnol.

[CR13] Gerba CP, Betancourt WQ (2017). Viral aggregation: impact on virus behavior in the environment. Environ Sci Technol.

[CR14] Jezek J, Chen D, Watson L, Crawford J, Perkins S, Tyagi A, Jones-Braun L (2009). A heat-stable hepatitis B vaccine formulation. Human Vaccines.

[CR15] Jiang H, Wang D, Wang J, Zhu S, She R, Ren X, Tian J, Quan R, Hou L, Li Z, Chu J, Guo Y, Xi Y, Song H, Yuan F, Wei L, Liu J (2019). Induction of porcine dermatitis and nephropathy syndrome in piglets by infection with porcine circovirus type 3. J Virol.

[CR16] Kim HJ, Kim HJ (2017). Current status and future prospects for human papillomavirus vaccines. Arch Pharm Res.

[CR17] Kim HJ, Kim HJ (2017). Yeast as an expression system for producing virus-like particles: what factors do we need to consider?. Lett Appl Microbiol.

[CR18] Kim HJ, Cho SY, Park MH, Kim HJ (2018). Comparison of the size distributions and immunogenicity of human papillomavirus type 16 L1 virus-like particles produced in insect and yeast cells. Arch Pharm Res.

[CR19] Kim SC, Nazki S, Kwon S, Juhng JH, Mun KH, Jeon DY, Jeong CG, Khatun A, Kang SJ, Kim WI (2018). The prevalence and genetic characteristics of porcine circovirus type 2 and 3 in Korea. BMC Vet Res.

[CR20] Kissmann J, Ausar SF, Foubert TR, Brock J, Switzer MH, Detzi EJ, Vedvick TS, Middaugh CR (2008). Physical stabilization of Norwalk virus-like particles. J Pharm Sci.

[CR21] Klaumann F, Dias-Alves A, Cabezon O, Mentaberre G, Castillo-Contreras R, Lopez-Bejar M, Casas-Diaz E, Sibila M, Correa-Fiz F, Segales J (2019). Porcine circovirus 3 is highly prevalent in serum and tissues and may persistently infect wild boar (Sus scrofa scrofa). Transbound Emerg Dis.

[CR22] Li X, Qiao M, Sun M, Tian K (2018). A duplex real-time PCR assay for the simultaneous detection of porcine circovirus 2 and Circovirus 3. Virol Sin.

[CR23] Liu LJ, Suzuki T, Tsunemitsu H, Kataoka M, Ngata N, Takeda N, Wakita T, Miyamura T, Li TC (2008). Efficient production of type 2 porcine circovirus-like particles by a recombinant baculovirus. Arch Virol.

[CR24] Madson DM, Opriessnig T (2011). Effect of porcine circovirus type 2 (PCV2) infection on reproduction: disease, vertical transmission, diagnostics and vaccination. Anim Health Res Rev.

[CR25] Mattle MJ, Crouzy B, Brennecke M, Wigginton KR, Perona P, Kohn T (2011). Impact of virus aggregation on inactivation by peracetic acid and implications for other disinfectants. Environ Sci Technol.

[CR26] Nainys J, Lasickiene R, Petraityte-Burneikiene R, Dabrisius J, Lelesius R, Sereika V, Zvirbliene A, Sasnauskas K, Gedvilaite A (2014). Generation in yeast of recombinant virus-like particles of porcine circovirus type 2 capsid protein and their use for a serologic assay and development of monoclonal antibodies. BMC Biotechnol.

[CR27] Noad R, Roy P (2003). Virus-like particles as immunogens. Trends Microbiol.

[CR28] Palinski R, Pineyro P, Shang P, Yuan F, Guo R, Fang Y, Byers E, Hause BM (2017). A novel porcine circovirus distantly related to known circoviruses is associated with porcine dermatitis and nephropathy syndrome and reproductive failure. J Virol.

[CR29] Raghunandan R (2011). Virus-like particles: innate immune stimulators. Expert Rev Vaccines.

[CR30] Sarker S, Terrón MC, Khandokar Y, Aragão D, Hardy JM, Radjainia M, Jiménez-Zaragoza M, de Pablo PJ, Coulibaly F, Luque D, Raidal SR, Forwood JK (2016). Structural insights into the assembly and regulation of distinct viral capsid complexes. Nat Commun.

[CR31] Shen H, Liu X, Zhang P, Wang L, Liu Y, Zhang L, Liang P, Song C (2018). Genome characterization of a porcine circovirus type 3 in South China. Transbound Emerg Dis.

[CR32] Shi L, Sanyal G, Ni A, Luo Z, Doshna S, Wang B, Graham TL, Wang N, Volkin DB (2005). Stabilization of human papillomavirus virus-like particles by non-ionic surfactants. J Pharm Sci.

[CR33] Stadejek T, Wozniak A, Milek D, Biernacka K (2017). First detection of porcine circovirus type 3 on commercial pig farms in Poland. Transbound Emerg Dis.

[CR34] Sun J, Wei L, Lu Z, Mi S, Bao F, Guo H, Tu C, Zhu Y, Gong W (2018). Retrospective study of porcine circovirus 3 infection in China. Transbound Emerg Dis.

[CR35] Tochetto C, Lima DA, Varela APM, Loiko MR, Paim WP, Scheffer CM, Herpich JI, Cerva C, Schmitd C, Cibulski SP, Santos AC, Mayer FQ, Roehe PM (2018). Full-genome sequence of porcine circovirus type 3 recovered from serum of sows with stillbirths in Brazil. Transbound Emerg Dis.

[CR36] Wu PC, Lin WL, Wu CM, Chi JN, Chien MS, Huang C (2012). Characterization of porcine circovirus type 2 (PCV2) capsid particle assembly and its application to virus-like particle vaccine development. Appl Microbiol Biotechnol.

[CR37] Wu P-C, Chen T-Y, Chi J-N, Chien M-S, Huang C (2016). Efficient expression and purification of porcine circovirus type 2 virus-like particles in *Escherichia coli*. J Biotechnol.

[CR38] Zhang Y, Wang Z, Zhan Y, Gong Q, Yu W, Deng Z, Wang A, Yang Y, Wang N (2016). Generation of *E. coli*-derived virus-like particles of porcine circovirus type 2 and their use in an indirect IgG enzyme-linked immunosorbent assay. Arch Virol.

[CR39] Zhang S, Wang D, Jiang Y, Li Z, Zou Y, Li M, Yu H, Huang K, Yang Y, Wang N (2019). Development and application of a baculovirus-expressed capsid protein-based indirect ELISA for detection of porcine circovirus 3 IgG antibodies. BMC Vet Res.

[CR40] Zheng S, Wu X, Zhang L, Xin C, Liu Y, Shi J, Peng Z, Xu S, Fu F, Yu J, Sun W, Xu S, Li J, Wang J (2017). The occurrence of porcine circovirus 3 without clinical infection signs in Shandong Province. Transbound Emerg Dis.

